# Microendoscopic calcium imaging of the primary visual cortex of behaving macaques

**DOI:** 10.1038/s41598-021-96532-z

**Published:** 2021-08-23

**Authors:** Mineki Oguchi, Jiang Jiasen, Toshihide W. Yoshioka, Yasuhiro R. Tanaka, Kenichi Inoue, Masahiko Takada, Takefumi Kikusui, Kensaku Nomoto, Masamichi Sakagami

**Affiliations:** 1 Brain Science Institute, Tamagawa University, Machida, Tokyo Japan; 2 School of Veterinary Medicine, Azabu University, Sagamihara, Kanagawa Japan; 3 Department of Neuroscience, Primate Research Institute, Kyoto University, Inuyama, Aichi Japan; 4 Department of Physiology, Dokkyo Medical University School of Medicine, Mibu, Tochigi Japan

**Keywords:** Neuronal physiology, Visual system

## Abstract

In vivo calcium imaging with genetically encoded indicators has recently been applied to macaque brains to monitor neural activities from a large population of cells simultaneously. Microendoscopic calcium imaging combined with implantable gradient index lenses captures neural activities from deep brain areas with a compact and convenient setup; however, this has been limited to rodents and marmosets. Here, we developed miniature fluorescent microscopy to image neural activities from the primary visual cortex of behaving macaques. We found tens of clear fluorescent signals from three of the six brain hemispheres. A subset of these neurons showed clear retinotopy and orientation tuning. Moreover, we successfully decoded the stimulus orientation and tracked the cells across days. These results indicate that microendoscopic calcium imaging is feasible and reasonable for investigating neural circuits in the macaque brain by monitoring fluorescent signals from a large number of neurons.

## Introduction

Macaque monkeys have long been used as experimental models to understand the neural mechanisms of the human brain. Extracellular recordings using microelectrodes have been traditionally used to record neural activity in the macaque brain. Recently, calcium imaging with fluorescence microscopy using acutely injected calcium-sensitive dye^1–4^ or genetically encoded calcium indicator^5–14^ has been applied. Calcium imaging allows the simultaneous recording of a number of neurons and thus is a powerful tool for understanding the function of local neural circuits at the mesoscopic scale. In primates, two-photon microscopy has been mainly used in calcium imaging studies to record neural activity from the cortical surface. In rodents, microendoscopic calcium imaging, which implants a gradient index (GRIN) lens into the brain and observes neural activity through a miniaturized fluorescent microscope, is also widely used, producing a number of innovative results concerning various brain areas, including deep neural nuclei^15–21^. Although there also has been a report on the application of microendoscopic calcium imaging to marmosets^22^, its application to macaque monkeys has not yet been reported.

Microendoscopy has several advantages over two-photon microscopy despite its lower temporal and spatial resolutions. In two-photon microscopy, adjusting the viewing angle to the observation plane is labor-intensive, and the animal must be completely immobilized against the experimental apparatus during an imaging session. For large animal species such as macaque monkeys, it is necessary to use an extra auxiliary fixture to achieve complete fixation of the animal’s head^7^. On the other hand, in microendoscopy, imaging data can be obtained simply by snapping the miniaturized microscope to a baseplate. The light weight and small size of the miniaturized microscope allow the observation of animals under free-moving conditions, although wireless data transmission will be necessary in the case of macaque monkeys, as has been done in large-scale microelectrode recordings^23^. Furthermore, by implanting GRIN lenses in multiple locations in the same brain, microendoscopy can be used for simultaneous recording of multiple locations. Given these advantages and potential utilities, it is useful to establish the viability of microendoscopy in the macaque brain.

In this study, we applied microendoscopic calcium imaging to macaque monkeys targeting the primary visual cortex (V1), where neurons can be easily activated by presenting visual stimuli. We incorporated the calcium indicator GCaMP6s into a mosaic adeno-associated virus vector packaged with AAV1 and AAV2 capsid protein (AAV2.1)^24^. We injected this virus into the bilateral V1 of three macaques and observed highly efficient infection in these regions. Prism lenses, which were equipped with a triangular prism at the tip of a rod lens, were implanted into the vector injection sites. Miniaturized microscopy through prism lenses revealed fluorescence changes in four hemispheres from two of the three monkeys and was able to identify dozens of neurons in three of these four hemispheres. For these two monkeys, Gabor patches with different orientations were presented in the peripheral field of view during fixation to examine the stimulus response. Observed V1 neurons had receptive fields at the corresponding positions on the retinotopic map and showed significant selective responses to specific orientations. We also successfully decoded the orientation of the visual stimulus and tracked the cells across days. In summary, we successfully recorded neuronal populations simultaneously in the macaque V1 using microendoscopy, and the observed cell populations showed response patterns consistent with the established V1 neuronal properties. These results indicate that microendoscopic calcium imaging is a powerful recording method for the macaque brain.

## Results

### Virus injection and prism lens implantation into the macaque V1

**Figure 1:**
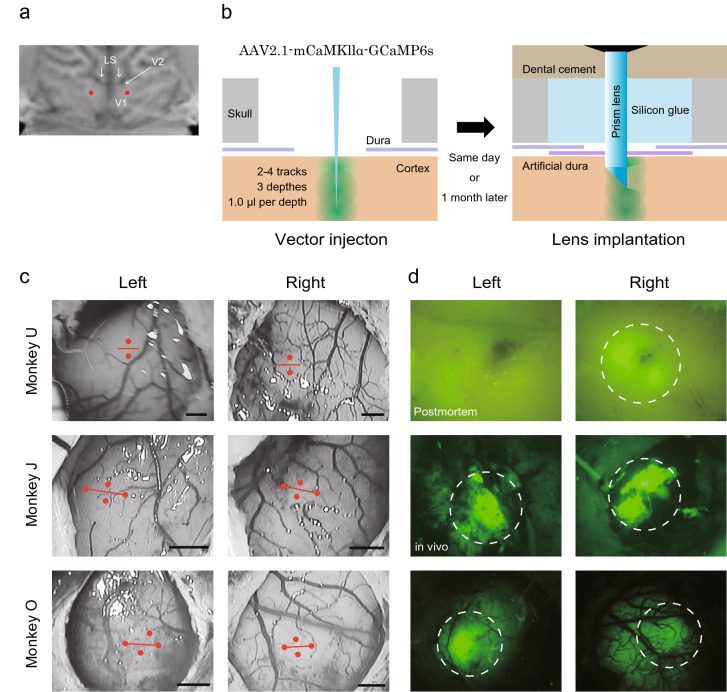
Vector injection sites and fluorescence expression on the cortical surface. (**a**) Representative target positions in the bilateral V1 (red dots). (**b**) Illustration of the virus injection and lens implantation procedures. AAV2.1 vector was injected at three different depths using a glass micropipette. At the same day or a month later, a prism lens was implanted into the location where the virus was injected. (**c**) Micrographs of the cortical surface showing the positions of virus injection and lens insertion in the left and right hemispheres of the three monkeys. Red dots and lines indicate the positions of virus injection and lens insertion, respectively. Scale bar: 1 mm. (**d**) Handheld fluorescent microscopy of virus injection positions postmortem (Monkey U) and in vivo (Monkey J and O). GCaMP expressions observed by the handheld fluorescent microscope were indicated within white dotted circles. LS, lunate sulcus; V1, primary visual cortex; V2, secondary visual cortex.

In this study, we used three macaque monkeys (Monkeys U, J, and O). To conduct microendoscopic calcium imaging from the bilateral V1 of the macaque brain during the monkeys’ performance of a behavioral task, we sequentially performed four surgeries: (1) attachment of a headpost to immobilize the monkeys in a monkey chair, (2) injection of the viral vector, (3) implantation of prism lenses, and (4) placement of the baseplate. We used GCaMP6s, which sum up several spikes in some temporal vicinity to the intensity of the fluorescence signal^25^, to obtain a strong intensity fluorescence signal in response to visual stimuli. PrismProbe (Inscopix, 1 mm diameter, 9.1 mm length) was used in this study to facilitate cortical penetration. PrismProbe was equipped with a triangular prism at the tip, and signals were collected from the side of the tip. We targeted the positions 12–14 mm from the midline to the lateral side and 4–6 mm from the lunate sulcus to the ventral side in V1 (Fig. 1a). This location is known to have a receptive field at 4° to 8° (visual angle) from the fovea and approximately − 45° in the lower quadrant of the visual field^26–29^.

For vector injection surgery (Fig. 1b), a 10-mm circular craniotomy was performed at the targeted locations, and an incision made in the dura mater was widened to expose the cortical surface. Vascularity was observed with a surgical microscope, and viral vectors were injected using glass micropipettes in positions that avoided large blood vessels. In Monkey U, the viral vector was injected into the tracks 0.3 mm dorsal and ventral from the center of the position where the prism lens was planned to be inserted. In Monkeys J and O, in addition to these two tracks, the viral vector was injected into another two tracks, which were 0.5 mm lateral and medial from the center of the lens position (Fig. 1c). In each track, 1.0 µL vector solution was injected at three different depths (Fig. 1b). Monkey U underwent vector injection and lens implantation surgeries on the same day. On the other hand, the craniotomy sites of Monkeys J and O were closed after vector injection. Subsequently, surgeries for lens implantation were performed after approximately 1 month to allow the expression of GCaMP6s. To determine the sites for lens implantation in Monkey J and O, the positions of viral vector injection were identified based on vascularity. Strong fluorescence signals were confirmed by handheld fluorescence microscopy at all four injection sites (Fig. 1d). Then, a scalpel blade was used to make a cut on the cortical surface in advance, and the lens was inserted through the cut and advanced 0.5 mm away from the position where the prism tip completely entered the cortex. The observation plane of the lens (1 mm × 1 mm) was set to face the dorsal side. Handheld fluorescence microscopy was also applied to the postmortem brain of Monkey U, showing a strong fluorescence signal around the lens trace in the right hemisphere, but not in the left hemisphere (Fig. 1d). After a sufficient recovery period after lens implantation, base plates were placed to mount the miniature fluorescent microscope.

**Figure 2:**
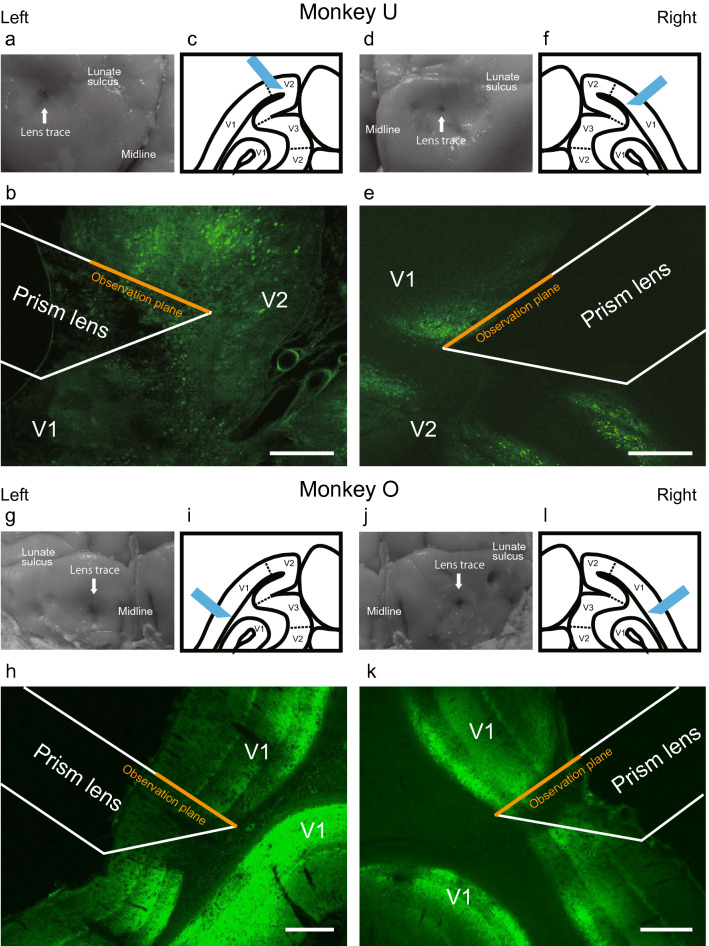
Immunohistochemistry and lens position. (**a**) Lens trace in the postmortem brain (left hemisphere) of Monkey U. White arrow indicates the hole where the lens punctured the brain. (**b**) 2D reconstruction of the position of the prism lens depicted on a micrograph of an immunostained brain section. Scale bar: 500 µm. (**c**) Illustration of lens position based on (**b**). (**d**–**l**) The corresponding figures to (**a**–**c**) for the right hemisphere of Monkey U and the left and right hemispheres of Monkey O. V1, primary visual cortex; V2, secondary visual cortex, V3, third visual cortex.

Immunohistochemical staining was performed on Monkey U and O to confirm GCaMP expression and lens positions after the completion of the imaging sessions. In Monkey U, we observed a high frequency of GCaMP expression in the V1 of the right hemisphere, and the population of GCaMP-positive cells was located close to the observation plane of the lens (Fig. 2d–f). The left hemisphere showed a similarly high frequency of GCaMP expression, but its position was found in V2 close to the lunate sulcus, and the population of GCaMP-positive cells was located slightly away from the observation plane (Fig. 2a–c). In Monkey O, a high frequency of GCaMP expression was observed in the wide V1 areas of both the left and right hemispheres, and the observation plane of the lenses was located in V1 close to GCaMP-positive cells (Fig. 2g–l). The lens insertion position in the left hemisphere was slightly closer to the midline than in the right hemisphere (Fig. 2g, j). As will be discussed in the next section, fluorescent signals with cellular-level resolution were observed in the right V1 of Monkey U and in the bilateral V1 of Monkey O, whereas only vague and diffused signals were observed in the left V1 of Monkey U. The success and failure of fluorescence observations in these hemispheres were consistent with the relationship between the lens positions and locations of GCaMP expression.

### Specific receptive fields of the detected V1 neurons

**Figure 3:**
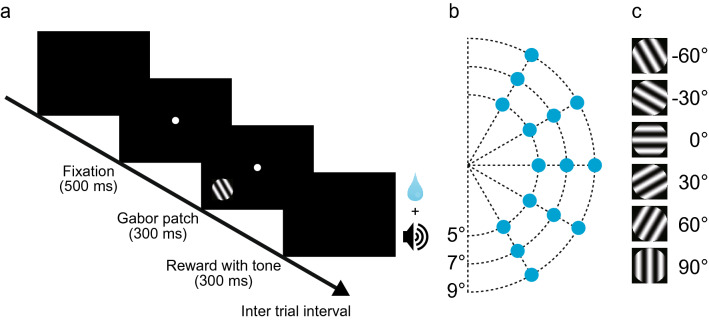
Fixation task with a Gabor patch presentation. (**a**) Time sequence of the fixation task with a peripheral Gabor patch presentation. After the monkey fixated on the central fixation point, a Gabor patch was briefly presented in its periphery. If the monkey kept fixated, a drop of water was given as a reward. (**b**) Blue circles indicate positions in the right visual field where the stimulus was presented in sessions to identify receptive fields for neurons recorded from the left hemisphere. (**c**) Six orientations of the Gabor patch presentation used in this study.

The monkeys were trained to perform a fixation task with a brief presentation of a peripheral visual stimulus (Fig. 3a). During this task, the monkeys were required to maintain fixation at the central fixation point (FP). A Gabor patch was briefly presented at the periphery of the field of view 500 ms after the start of fixation. If the monkeys were successful in continuing to fixate on the FP, a correct sound and a drop of water as a reward were delivered. We first conducted sessions to identify receptive fields. During these sessions, Gabor patches were sequentially presented at 15 locations in the field of view opposite to the recorded hemisphere (Fig. 3b). In subsequent sessions, Gabor patches were presented in a fixed position where the receptive field was identified. In all of these sessions, Gabor patches had six different orientations, and they were presented repeatedly in sequence (Fig. 3c).

After injection of the virus vector and installation of the lens and base plate following task training, microendoscopic calcium imaging was performed from the bilateral V1 of the three monkeys under awake conditions. We observed fluorescence dynamics in the bilateral V1 of two out of the three monkeys (four hemispheres of Monkey U and O). In the right hemisphere of Monkey U and both hemispheres of Monkey O, multiple fluorescence signals with cellular-level resolution were observed. Only a vague signal of change was observed in the left hemisphere of Monkey U and no fluorescence changes were detected in either hemisphere of Monkey J.

**Figure 4:**
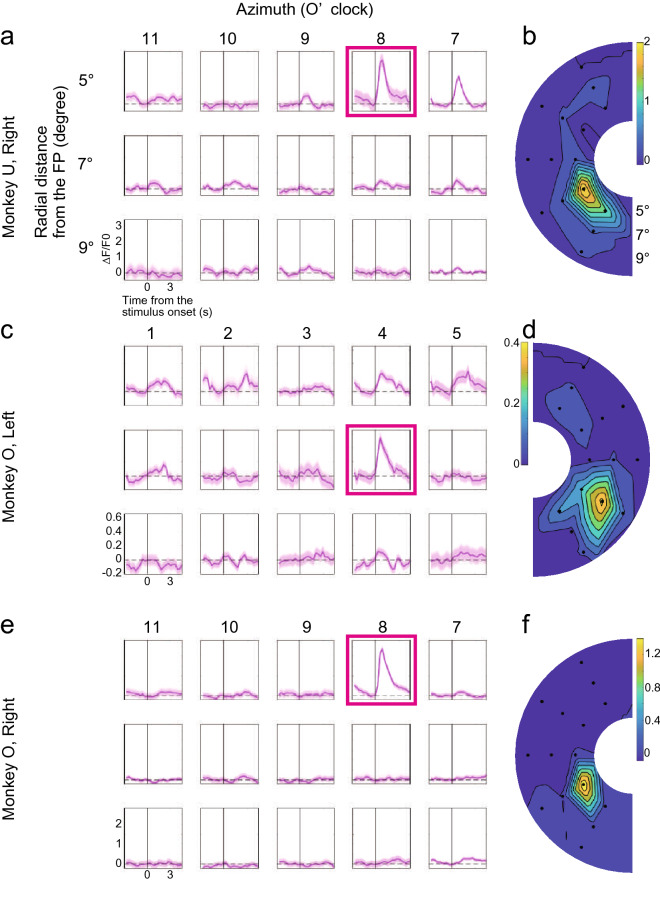
Receptive fields of recorded V1 cells. (**a**) Averaged stimulus responses of a representative ROI at different stimulus positions for the right hemisphere of Monkey U. These panels are arranged according to the azimuth (columns) and eccentricity (rows) of the stimuli. Error areas: standard error of the mean. (**b**) Position of a receptive field depicted by a linear model curve fitting. Color indicates normalized signal strength (ΔF/F0). (**c**–**f**) The corresponding figures to (**a**) & (**b**) for the imaging data recorded from the left and right hemispheres of Monkey O.

We then conducted recording sessions in Monkeys U and O to identify the receptive fields of neural populations (see “Methods” section). We used constrained nonnegative matrix factorization for microendoscopic data (CNMF-E)^30^ to detect putative neurons as regions of interest (ROIs) (see “Methods” section). Because neurons observed through each lens showed almost similar receptive fields, the analysis here used a time series of calcium dynamics from one representative ROI for each hemisphere that showed stimulus responses to many orientations. In the right hemisphere of Monkey U, stimulus responses were strongest at 5° eccentricity at 8 o’clock (Fig. 4a, b). In the left hemisphere of Monkey O, stimulus responses were strongest at 7° eccentricity at 4 o’clock (Fig. 4c, d), while in the right hemisphere, stimulus responses were strongest at 5° eccentricity at 8 o’clock (Fig. 4e, f). These receptive fields were in the vicinity where we expected them to be located based on the known retinotopic map in macaque V1^26–29^.

### Detecting putative neurons

We then performed recording sessions to examine the orientation selectivity of the detected putative neurons by presenting Gabor patches to the identified receptive fields in each of the three hemispheres. Each of the six different orientations was presented 20 times, and the orientation selectivity index (OSI) was calculated using the data obtained from each detected putative neuron (see “Methods” section). The OSI is the highest (OSI = 1) when the ROI responds only to one orientation and not to the other. The response strength to each orientation was also used to calculate the preferred orientation of the ROI.

**Figure 5:**
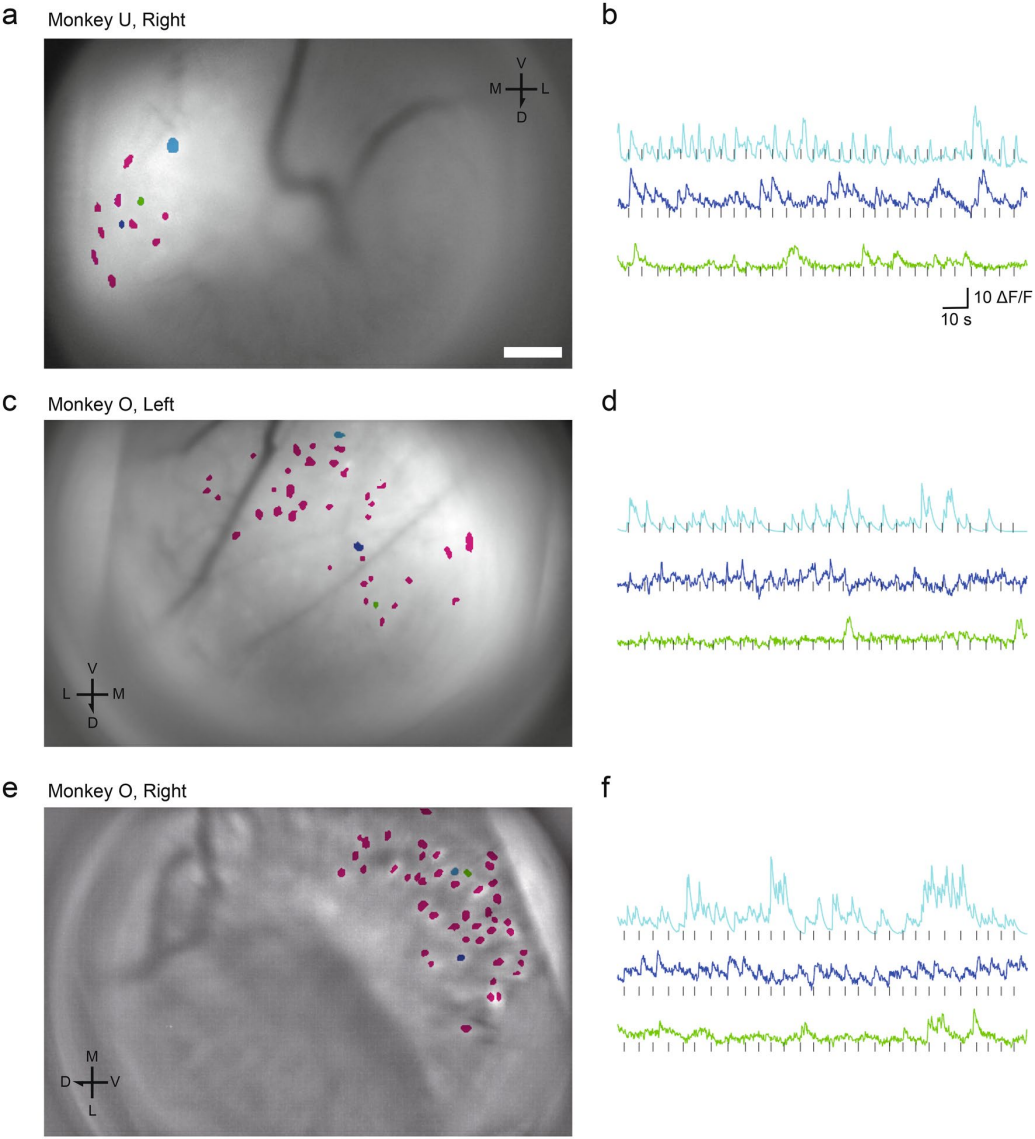
ROI detection and orientation tuning. (**a**) Colored dots indicate ROIs (putative neurons) detected from an imaging data recorded from the right hemisphere of monkey U. The background image is a Z projection using the maximum intensity of the imaging data. Scale bar: 100 µm. (**b**) Examples of time-series data of normalized fluorescence signals. Each colored line corresponds to ROIs filled with similar color in (**a**). Short black vertical lines represent timings of the stimulus onset. (**c**–**f**) The corresponding figures to (**a**) and (**b**) for the imaging data recorded from the left and right hemispheres of Monkey O. V, ventral; D, dorsal; M, medial; L, lateral.

In a representative session recorded from the right hemisphere of Monkey U, 12 ROIs were detected in the medial side of the field of view (Fig. 5a, Supplementary Movie 1). For Monkey O, 37 and 45 ROIs were detected in the ventromedial side of the field of view in a session recorded from the left (Fig. 5c, Supplementary Movie 2) and right (Fig. 5e, Supplementary Movie 3) hemispheres, respectively. Figure 5b, d, f shows the time series of the fluorescence signals of typical ROIs in Fig. 5a, c, e, respectively. Many of these putative neurons were significantly responsive to visual stimuli presented to the receptive field (75/94, 79.8%).

### Orientation tuning in the detected ROIs

**Figure 6:**
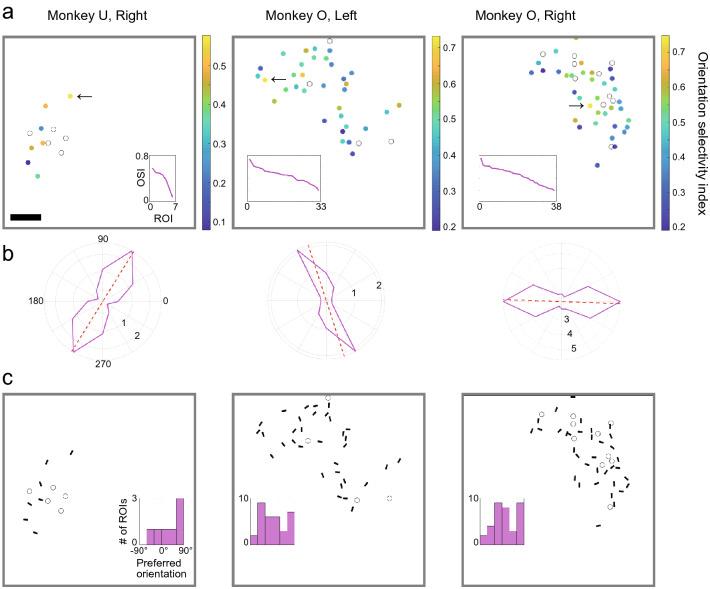
Maps of orientation selectivity and preferred orientation. (**a**) Color maps of OSI values of detected ROIs. The recording sessions used are the same with those used in Fig. 5. Filled circles indicate ROIs with stimulus responses, and open circles indicate ROIs without stimulus responses. The color of each filled circle represents the OSI value shown in the color bar. Inset: A graph showing the OSI values from largest to smallest in the corresponding map. Scale bar: 100 µm. (**b**) Polar coordinate displays of signal intensity for each orientation calculated from the time-series data of the ROI indicated by the black arrows in (**a**). Radial distance means normalized signal strength (ΔF/F0). The dashed red line represents the preferred orientation of this putative neuron. (**c**) The orientation of each black bar represents the preferred orientation of the corresponding ROI with stimulus responses. Open circles indicate ROIs without stimulus responses. Inset: A histogram showing the distribution of preferred orientation in the corresponding map.

To examine whether the detected ROIs show selectivity for a specific orientation, we calculated the OSI value for each ROI. The distribution of OSIs presented in Fig. 6a shows that they vary from large to small (Fig. 6a, inset). Figure 6b shows the polar coordinate displays of the stimulus responses of the ROIs with the largest OSI values among those detected from each hemisphere. In addition, we calculated the preferred orientation of each ROI as the direction of the vector sum of the responses to six orientations of the visual stimulus. The distributions of preferred orientations are shown in Fig. 6c. Except in the left hemisphere of Monkey U, where the number of ROIs was relatively small, the distributions of preferred orientations were not highly biased toward any particular orientation (Fig. 6c, inset).

### Decoding orientations of presented stimuli from imaging data

**Figure 7:**
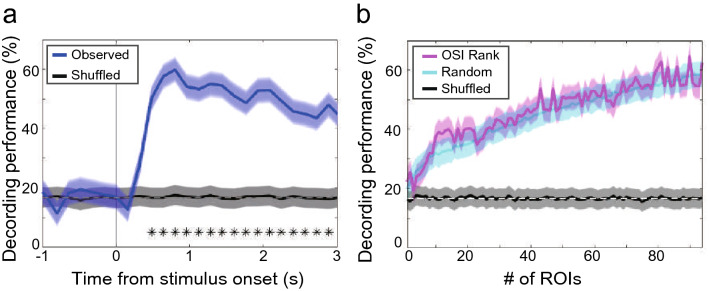
Decoding the orientation of presented stimuli. (**a**) Orientation decoding performance before and after stimulus presentation using ROIs detected from V1 in the three hemispheres. The blue and black lines represent the decoding performances using the observed and shuffled data, respectively. Asterisks indicate significant differences between the results from observed and shuffled data (α < 0.00001). Error areas: standard error of the mean. (**b**) Performance when the number of ROIs used for decoding is increased. Magenta represents the case where ROIs are selected from high to low OSI values. Cyan represents the case where ROIs are randomly selected from observed data. Black represents the case where ROIs are randomly selected from shuffled data. Error areas: standard error of the mean.

Next, we calculated the decoding performance using a support vector machine to determine whether we could decode the orientation of the visual stimuli from calcium dynamics data obtained by microendoscopy. In this analysis, data from the representative sessions for each of the three hemispheres were merged (94 ROIs in total). We examined the decoding performance over time by a sliding window using time series data aligned to stimulus onset and found that the performance increased after stimulus onset and became significantly higher than shuffled data at approximately 500 ms (Fig. 7a). Then, to investigate how decoding performance after stimulus onset is affected by the number of ROIs, we gradually increased the number of ROIs to be used in this analysis. We used both the random selection of ROIs from 94 ROIs and the addition of ROIs in order, starting with the one with the highest OSI. As a result, the decoding performance of the random selection method increased gradually as the number of ROIs increased. The decoding performance of the highest-to-lowest method was not significantly different from that of the random selection method (Fig. 7b). Additionally, the peaks in decoding performance came at the points where all or most ROIs were used, indicating that information from ROIs other than the high-value OSI is also necessary to obtain high decoding performance.

### Cell tracing across days

**Figure 8:**
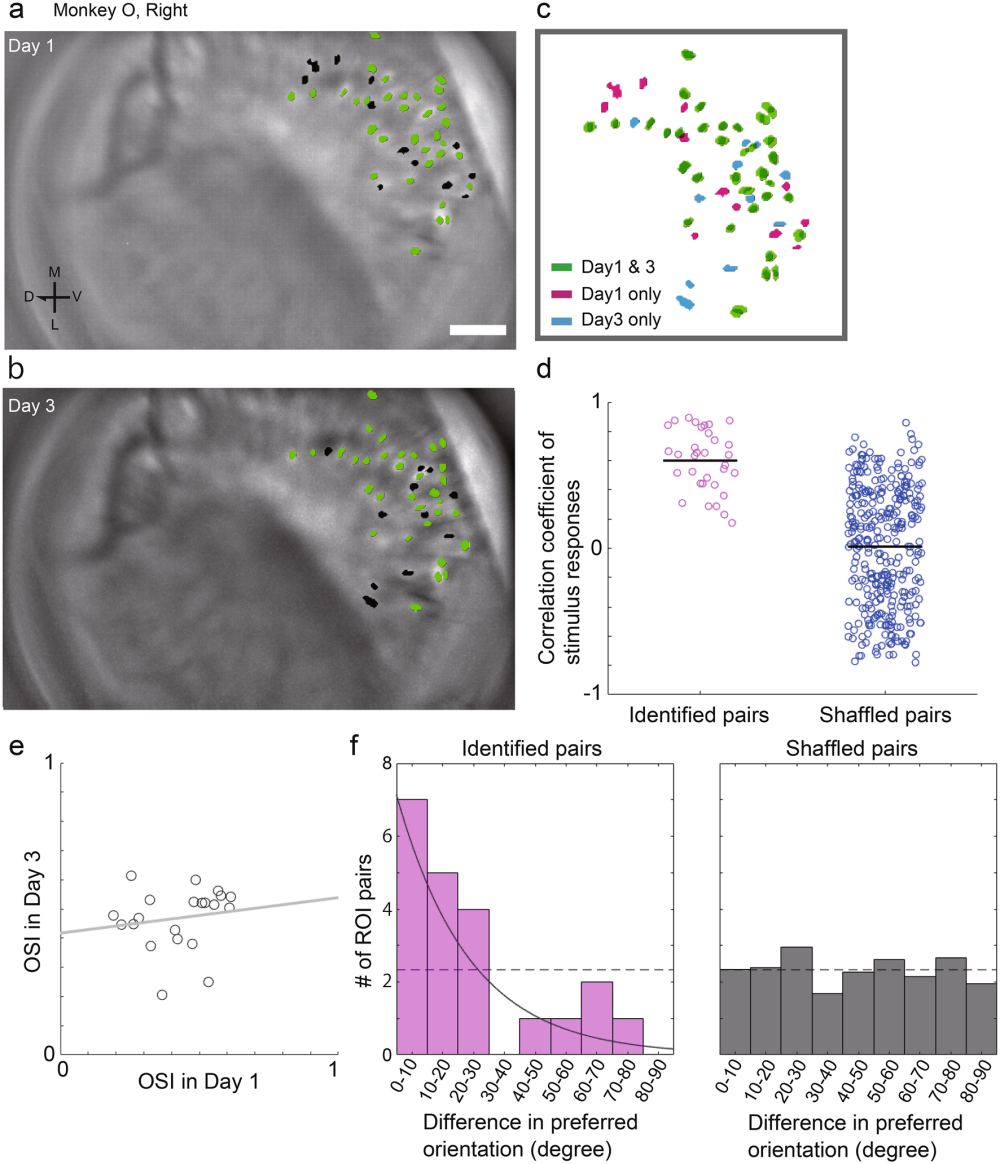
Cell tracing across days. (**a**) The same ROI distribution map with Fig. 5e. Green dots are identified as the same-colored dots in Day3. Black dots are ROIs that were not identified. (**b**) The ROI distribution map of Day3, which is two days after Day1. Green dots are identified as the same-colored dots in Day1. Black dots are ROIs that were not identified. Scale bar: 100 µm. (**c**) ROIs identified across days, only on Day1, and only on Day3 are shown in green, magenta, and cyan, respectively. (**d**) Correlation coefficients of fluorescence signals after stimulus presentation (0–2000 ms) between ROI pairs identified on Days 1 and 3 averaged over different orientations. The magenta dots represent identified pairs, and the blue dots represent shuffled pairs. (**e**) Scatter plot of OSI on Day1 and Day3 for identified pairs. The dots represent each identified ROI. (**f**) Histogram of the absolute values of the difference in preferred orientation between ROI pairs identified on Days 1 and 3. Zero means that the ROI showed the same value of preferred orientation on both days. The magenta bars in the left panel are the actual observed data, and the gray bars in the right panel are the shuffled data. The horizontal dashed line represents the chance level. The solid line represents the exponential curve fitted to the observed data. V, ventral; D, dorsal; M, medial; L, lateral.

Using microendoscopic calcium imaging, the same cells can be observed across days by implanting a GRIN lens deep in the brain^15, 16, 19, 31–33^. We were able to maintain observations at cellular-level resolution across days for the right hemisphere of Monkey O. Here, we used imaging data from Days 1 and 3 to track the detected ROIs. We applied a cell tracking method with a probabilistic model using centroid distance and spatial correlation between the footprints of the ROIs^34^ (see “Methods” section). As a result, we were able to track a large portion of the cells detected on Day1 in the imaging data from Day3 (Fig. 8a–c; 33/45 ROIs, 73.3%).

To determine whether the identified ROIs had similar cellular characteristics, we first calculated the Pearson correlation of the stimulus–response between the two recording sessions. We calculated the correlation of the fluorescence time-series data (0–2000 ms after the stimulus onset) for each orientation between the identified ROIs, and the average of the correlation coefficients was obtained for each pair. Examples of the top three pairs of correlation coefficients are shown in Supplementary Fig. 1a. The correlation coefficients for the identified pairs were significantly higher than those for the shuffled pairs (Fig. 8d; t(361) = 8.20, *p* = 4.29E-15, two-sample t-test). We then calculated the correlation between the OSI values of the identified pairs. Only ROIs that showed a significant stimulus–response on both days (Days 1 and 3) were used in this analysis (21/33 ROIs). However, no significant correlation was found (Fig. 8e; r = 0.158, *p* = 0.495, Pearson correlation). Finally, we calculated the difference in preferred orientation between the two recording sessions (Fig. 8f). The results showed that the proportion of ROIs that had small differences of less than 10° as the largest, and the proportion of ROIs decreased as the differences increased. When the ROI pairs were shuffled, the proportion of differences was uniformly distributed. Therefore, we performed a uniformity test on the identified ROIs. The distribution of observed values was significantly different from the uniform distribution (*p* = 0.019, two-sample Kolmogorov–Smirnov test). However, the distribution was not significantly different from the fitted exponential distribution (*p* = 0.958), suggesting that many of the tracked cells retained their response properties.

As an additional analysis, we performed the same cell tracing between two consecutive recordings on the same day (Rec1 and Rec2 on Day1). As a result, 84.4% of ROIs in Rec1 were tracked in Rec3 (Supplementary Fig. 2a–c; 37/45 ROIs, 84.4%). The Pearson correlation of stimulus–response between the identified pairs was significantly higher than that of the shuffled pairs (Supplementary Fig. 1b and 2d; t(416) = 8.86, *p* = 2.41E-17, two-sample t-test). While the correlation between the OSI values was not significant (Supplementary Fig. 2e; 26/37 ROIs, r = 0.149, *p* = 0.469, Pearson correlation), the difference in preferred orientation between identified pairs was biased toward zero (Supplementary Fig. 2f), as was the case across days. The distribution was significantly different from a uniform distribution (*p* = 0.019, two-sample Kolmogorov–Smirnov test), but not significantly different from an exponential distribution (*p* = 0.603).

## Discussion

Microendoscopic calcium imaging is rapidly gaining widespread use in systems neuroscience, particularly in rodents, and has generated numerous new insights through observations of various brain regions, including deep nuclei. However, this technique has not yet been applied to macaque monkeys, despite it being a powerful model for elucidating human brain function. To establish the feasibility of microendoscopic calcium imaging in the macaque brain, we tested it with bilateral V1 as a target area. As a result, we detected fluorescent signals with cellular resolution in response to visual stimuli in three of the six hemispheres used in this study. The detected neurons had receptive fields in locations consistent with the known retinotopic map and showed tuning to a specific orientation, which are characteristics of the V1 cells. We also successfully decoded the orientation of the stimuli presented to the monkeys and tracked the cells across days using the calcium dynamics data. These results show that microendoscopic calcium imaging is an effective observation method, even in macaque monkeys.

The properties of macaque V1 neurons have traditionally been explored using electrophysiological techniques. The results of our study are consistent with those of previous studies in many respects. It has been shown that the eccentricity of the receptive field is greater in regions closer to the midline on the V1 surface^26–29^. Consistent with this, in Monkey O, the lens injection position in the left hemisphere was closer to the midline than that in the right hemisphere, and the eccentricity of the receptive field was greater in the left hemisphere than in the right hemisphere. The magnification factor (the cortical surface distance between two points representing visual field positions 1° apart) was found to be 1–2 mm/degree around the eccentricity (5°–7°) of the receptive field we identified^27–29^. Based on this, the maximum change in the receptive field position across the field of view of the prism lens we used is expected to be less than 1°. This is consistent with our observation that the fluorescence signals in the field of view recorded by each lens had almost the same receptive field. V1 cells with similar orientation selectivity are known to assemble to form a columnar structure^35^. Previous studies using two-photon microscopy have detected the columnar structure in macaque V1 by observing the cortical surface from the top^7, 13^. In this study, we used prism lenses to observe the cortex from the side. Therefore, we were not able to observe the column structure, but we were able to observe the cells in the deep layers of V1. Previous studies have shown that input layers, such as 4C and 6, and output layers, such as 2/3 and 5, have different cellular properties^36–39^. The orientation selectivity was shown to be relatively variable in the input layer^37^, consistent with our observations. However, because of the relatively small area and a limited number of cells we observed, it is difficult to determine from which layers we recorded the fluorescent signals. It is also known that the preferred orientation changes smoothly across layers along the direction perpendicular to the cortical surface, but the degree of change is not constant and is highly variable^39^. This is consistent with our result that there is no simple regularity in the distributions of the preferred orientation.

We succeeded in tracking the cells across days and found similarities in the time series of stimulus-responses and orientation preferences among the identified cells. However, we did not find any correlation in the orientation selectivity. This may be due to the large variation in the orientation selectivity of the observed cells.

We performed viral vector injection and prism lens implantation in a total of six hemispheres of three monkeys. Although handheld fluorescence microscopy and immunohistochemical staining showed GCaMP6s expression in all six hemispheres, calcium dynamics at the cellular-level resolution were successfully observed in three of them. Postmortem fluorescent staining of the brain of Monkey U showed that the expression of GCaMP6s was close to the observation plane of the lens in the right hemisphere, where neurons were visible with good resolution, while the expression of GCaMP6s was slightly away from the observation plane in the left hemisphere, where only diffused signals were visible with poor resolution. This suggests that the success or failure of imaging depends primarily on how the lens can be implanted in the vicinity of the expressed calcium indicators. Future technical improvements in both wider viral infection and precise lens implantation will be needed to increase the detection rate of calcium signals.

In this study, we reproduced the well-established characteristics of V1 cells, which include receptive field and orientation tuning, using time-series data of the detected ROIs. We also successfully decoded the orientation of visual stimuli from microendoscopic data, despite the relatively low temporal resolution. These results demonstrate the potential of microendoscopic calcium imaging for elucidating the functions of brain areas other than V1, such as the prefrontal cortex. In particular, it is suitable for investigating the dynamics of local neural circuits in long-term learning. Moreover, we successfully tracked many cells across days. Other rodent studies have succeeded in tracing the same cells over weeks with high recording quality using microendoscopy^31^. With this method, it is possible to examine how the response properties of individual neurons and the organization pattern of neural circuits change during the learning process. Furthermore, one of the advantages of calcium imaging is the minimal bias in the sampling of cells to be recorded. Even low-frequency cells that would be discarded by extracellular recording can still be recorded if they are within the field of view and express sufficient amounts of calcium indicators. When such cells change their response properties as a result of learning, it may be possible to review past data and retrospectively examine the same cells. Thus, microendoscopic calcium imaging allows for multifaceted analysis of the dynamics of local neural circuits in non-human primates.

## Methods

### Subjects

Three male Japanese monkeys (*Macaca fuscata*) were used as experimental subjects (Monkey U, 12.8 kg; Monkey J, 8.2 kg; and Monkey O, 8.6 kg). All experimental protocols in this study were approved by the Animal Care and Use Committee and the Safety Committee for Genetic Modification Research at Tamagawa University and were in accordance with the National Institutes of Health’s Guide for the Care and Use of Laboratory Animals and with the ARRIVE guidelines. The monkeys were kept in individual primate cages in an air-conditioned room where food was available ad libitum. The body weight and appetite of the monkeys were checked, and vegetables and fruits were provided daily. In the breeding room, many cages face each other to allow the monkeys to see and hear each other.

### Virus vector

The AAV2.1-CaMKIIα-GCaMP6s vector (6.0 × 10^13^ genome copies per mL) was produced by the helper-free triple transfection procedure and was purified by affinity chromatography (GE Healthcare). Viral titers were determined by quantitative PCR using TaqMan technology (Life Technologies). The transfer plasmid (pAAV- CaMKIIα-GCaMP6s-WPRE) was constructed by inserting the mouse CaMKIIα promoter, GCaMP6s gene, and WPRE sequence into an AAV backbone plasmid (pAAV-CMV, Stratagene).

### Surgery

To perform microendoscopic calcium imaging, we performed a series of surgeries on each monkey: (1) installation of a head holder; (2) injection of viral vectors once in each of the left and right hemispheres, (3) implantation of prism lenses, and (4) installation of a baseplate to hold the miniaturized microscope. In each surgery, the monkeys were anesthetized by intramuscular injections of ketamine hydrochloride (10 mg/kg) and xylazine (1 mg/kg) and maintained under general anesthesia with isoflurane (1.0–2.0%). In the head holder surgery, after the skull was exposed, a head holder placed at the midline and ear bar zero was attached to the dental acrylic head implant, which was fastened to the skull by acrylic screws.

After finishing the task training, the second and third surgeries were performed to inject the viral vector into the V1 of the monkeys. The dental cement around the injection site, which was determined based on MRI images, was removed, and the skull was craniotomized into a 10-mm diameter circle. Then, the dura at the craniotomy site was cut longitudinally and sutured to widen it on both sides to expose the cortex. The viral vector injection site was determined to avoid large blood vessels, and the virus was injected using a glass micropipette connected to a 10-µL Hamilton syringe. The micropipette was manually lowered using a micromanipulator (SM-11, Narishige, Tokyo, Japan). We injected 1.0 µL vector solution at depths of 2.5, 1.5, and 0.5 mm from the cortical surface. Injections were made using an electric syringe pump (Legato210P, KD Scientific, MA, USA) at a rate of 0.2 µL/min. After injection, we waited for 5 min to prevent diffusion and backflow. Two injections were made around the lens implantation site for Monkey U and four tracks for Monkey J and Monkey O. Viral vector injection and lens implantation were performed on the same day for Monkey U and on different days about a month apart for Monkeys J and O. After vector injection surgery for Monkeys J and O, an artificial dura was placed under the native dura, covered with medical silicone adhesive and sealed with dental cement.

For the prism lens implantation surgery performed on the monkeys, the vector-injected holes were first re-exposed, and the locations for the lens placement were determined based on the vascularity and fluorescence observation of the cortical surface using a handheld fluorescence microscope (Dino-Lite, Opto Science, Tokyo, Japan). The prism lens was inserted into the cortex with the observation plane placed on the dorsal side. Prior to lens insertion, a scalpel blade was used to make an incision on the cortical surface to ensure smooth insertion of the lens. The scalpel blade (No. 11) was pierced to the required depth (2 mm) and moved 1 mm parallel to the blade. The blade was then withdrawn and reattached in the opposite direction to make another incision. The prism lens was inserted in line with this incision and was advanced until the upper edge of the observation plane was completely hidden within the cortical tissue by repeatedly moving 1 mm forward and 0.5 mm backward. After the prism lens was advanced to a sufficient depth, an artificial dura mater was placed around the lens, medical silicone adhesive (Kwik-sil, World Precision Instruments, FL, USA) was poured, and the periphery of the lens was secured with dental cement. The top of the lens was covered with a small piece of plastic paraffin film and covered with another silicone adhesive (Kwik-cast, World Precision Instruments, FL, USA). Finally, a chamber with a lid was placed with dental cement to protect the lens from being touched by the monkeys.

One month after lens implantation, the final surgery was performed to place the baseplate. After adjusting the angle, the baseplate was slowly moved closer to the lens during observation using a miniaturized microscope. The baseplate was fixed at a position where the blood vessels observed from the lens were clearly visible. The dental cement securing the baseplate was mixed with black acrylic paint to prevent light exposure. Imaging sessions with the monkeys while they perform the fixation task were conducted after a sufficient recovery period.

### Behavioral task

The monkeys were trained in a fixation task using a Gabor patch presentation and performed in a dark, sound-attenuated room. The monkeys were seated in a primate chair in front of a 20-inch LCD monitor (2005FPW, Dell, TX, USA) with their heads fixed. The distance between their eyes and the display was 57 cm. A trial started with the onset of the central FP, which was a white circle with a 1.0° diameter on the monitor. Then, 500 ms after the monkeys started fixating at the FP, a Gabor patch was presented around the FP for 300 ms. If the monkeys continued to fix their eyes within a 3° fixation window around the FP, then a success tone (1000 Hz) and water (0.3 mL) were given. Otherwise, no reward or an error tone (200 Hz) was delivered. After the inter-trial interval (4000 ± 1000 ms), the next trial was started. During the sessions to identify the receptive field, Gabor patches were presented in sequence at viewing angles of 5°, 7°, and 9° from the FP, at azimuths 1, 2, 3, 4, and 5 o’clock (when recording from the left hemisphere), and at 11, 10, 9, 8, and 7 o’clock (when recording from the right hemisphere). At each position, a Gabor patch (3° in diameter, 1 cycle/degree) was presented sequentially at − 90°, − 60°, − 30°, 0°, 30°, and 60°. Once the receptive field was identified, the Gabor patches were presented at the same position as the identified receptive field position in the subsequent recording sessions. The task was controlled using the TEMPO system (Reflective Computing, MO, USA). The visual stimulus presentation for the monitor was programmed by a custom-made program using an application programming interface (OpenGL).

### Data acquisition

Calcium imaging data were acquired using a miniaturized fluorescence microscope (nVISTA 3.0, Inscopix, CA, USA) with 0.2–0.5 mW/mm^2^ of irradiance at 6–10 fps. Eye movement was monitored using an infrared camera system at a sampling rate of 240 Hz (Eye-Trac 6000, Applied Science Laboratories, MA, USA).

### Data analysis

#### ROI detection

The recorded imaging data were processed using the Inscopix Data Processing Software (Inscopix, CA, USA). For pre-processing, the recorded images were spatially downsampled by a factor of four, spatially filtered, and motion corrected. To extract cells showing fluorescence changes as ROIs, we used constrained nonnegative matrix factorization for microendoscopic data (CNMF-E^30^), which is a cell detection method based on a constrained matrix factorization approach and optimized for single-photon data obtained by microendoscopy. The resulting cell candidates were narrowed down by contrasting them with the video data. The resulting normalized time series data of the ROIs were then analyzed using custom codes in MATLAB (R2017b, MathWorks, MA, USA).

#### Retinotopy and orientation selectivity

To examine the receptive fields of the extracted cell populations, we used the data obtained by presenting six orientations of Gabor patches at each of the 15 peripheral stimulus presentation locations (12 trials per location for two rounds of stimulus presentation) contralateral to the recoded hemisphere. Because nearly identical receptive fields were shown in the cell populations observed from each lens, we used time series data from representative ROIs that responded to many of the six orientations. The obtained time series data of fluorescence intensity were normalized using the mean and standard deviation of the entire recording session. Only the data from successful trials were used in this and subsequent analyses. Data from each trial were aligned to the stimulus onset and combined into the data matrices for each stimulus position. The average intensity from − 500 to 0 ms before stimulus onset was subtracted to adjust the baseline. The Tukey–Kramer test was used for multiple comparisons of the fluorescent signals averaged over the post-stimulus period (500–1500 ms). The position with the strongest stimulus response among all positions and was significantly different from the other non-neighborhood positions was detected as the receptive field. To visualize the location of the receptive field, we performed 3D fitting using a linear model embedded in MATLAB's curve approximation tool.

Next, we used the data obtained by presenting Gabor patches (20 trials for each orientation) at the identified receptive fields to examine the orientation selectivity of the cell population. To investigate whether the detected putative neuron responded to the presented stimulus, we used the data from the pre-stimulus period (− 1000 ms to 0 ms from the stimulus onset) as a baseline and compared it with the mean signal intensity between the post-stimulus period (500–1500 ms) separately for each orientation. Multiple comparisons were performed using Bonferroni's method, and a putative neuron was considered stimulus-responsive if it was significantly different from the baseline (α = 0.05). To calculate the OSI, the peak signal intensity after stimulus presentation (500–1,500 ms) was used in the following function:

$$\mathrm{OSI}=\frac{\sqrt{{(\sum R\left(\theta \right)\mathrm{sin}(2\theta ))}^{2}+{(\sum R\left(\theta \right)\mathrm{cos}(2\theta ))}^{2}}}{\sum R(\theta )}$$

where *θ* represents the stimulus orientation, and *R(θ)* represents the intensity of the evoked response at *θ*. Because the signal can take a negative value, we added a constant to all response values to set the minimum response to zero^3^. We also calculated a negative OSI value for each ROI because some ROIs respond negatively to stimuli. The absolute values of positive and negative OSIs were compared, and the greater value was taken as the OSI for that ROI. The preferred orientation of each ROI was determined as the orientation of the vector sum calculated based on the peak signal intensities after stimulus presentation (500–1500 ms).

#### Decoding

We used time series data from the detected ROIs to decode the orientation of the Gabor patches presented in each trial. A multi-class error-correcting output code model consisting of a binary support vector machine was trained to decode the six orientations, which was performed by the fitcecoc function in MATLAB. The trained model was used to obtain error estimates for tenfold cross-validation, and the decoding performance was calculated from the classification loss. To calculate the time course of the decoding performance, we used a 500-ms sliding window moving by 166-ms step to the time series data aligned to the stimulus onset. For the control, we used the same procedure to obtain the decoding performance using shuffled labels for orientations of the stimuli and repeated the decoding procedure 100 times at each step. For each time-bin, two-tailed two-sample *t*-tests (α = 0.05) were used to determine whether there was a significant difference between the obtained decoding performance and that of the shuffled data. Next, to examine the change in decoding performance depending on the number of ROIs used, we either randomly selected *n* ROIs from a pool of ROIs and decoded the orientation of stimuli using them, or selected *n* ROIs in order of their OSI. For the control, we also used shuffled labels for the orientation of the stimuli to obtain the decoding performance for the randomly selected *n* ROIs. For the random selection and shuffled processes, we repeated the decoding procedure 100 times for each number of ROIs. In this analysis, we used the data from 500 to 1500 ms after stimulus onset.

#### Cell tracking across sessions

To track cells between different imaging data, we used a cell tracking method with a probabilistic model that uses the centroid distance and spatial correlation between footprints of the ROIs^30^, in which the fields of view obtained in the two imaging sessions were first aligned and the centroid distance between the centers of gravity of the ROI footprints and the Pearson correlation of footprint shapes were calculated. Then, probabilistic models for these features were fitted based on the Bayesian framework, and the ROI tracking scores were evaluated using a mixture model. ROI pairs that score above a threshold (default value: P_same_ = 0.5) are registered as identical.

To examine the similarity of the stimulus responses, Pearson correlations for the stimulus responses (0–2000 ms after the stimulus onset) at each orientation were calculated and its average over different orientations was compared to the data of shuffled pairs (10 times the number of tracked pairs) using a two-sample *t*-test. Pearson correlations were also calculated for the OSI values between tracked pairs. In addition, we calculated the differences in the preferred orientations in each session for the ROIs. For comparison, the difference in preferred orientations was also calculated for the shuffled pairs. This shuffling process was repeated 10,000 times. Histograms for each difference data were obtained by setting the bin width to 10°. For the shuffled data, the number of elements was divided by 10,000. Finally, we tested whether the distributions obtained from the actual ROI pairs differed from the uniform and exponential distributions using the two-sample Kolmogorov–Smirnov test (the former was set using theoretical values and the latter was set by fitting the actual data to an exponential curve with the fit function in MATLAB).

### Immunohistochemistry

Following the recording sessions, the monkeys were deeply anesthetized with an intravenous injection of sodium pentobarbital (70 mg/kg, i.v.) and transcardially perfused with 0.01 M PBS followed by 4% paraformaldehyde in 0.1 M phosphate buffer (pH 7.4). Brains were extracted and post-fixed in 4% paraformaldehyde overnight at 4 °C and cryoprotected with increasing gradients of sucrose (5%, 10%, and 20%). Frozen brains were then sliced into coronal sections at 40-µm thickness using a cryostat.

One in four successive sections was immunohistochemically stained. Free-floating sections were washed with PBS and permeabilized in PBS containing 0.3% Triton X-100 (PBST). After blocking for 1 h in 3% normal goat serum in PBST containing 1% bovine serum albumin (BSA-PBST), sections were incubated for 2 nights at 4 °C with a mouse anti-GFP antibody (1:250, Millipore, MA, USA) in BSA-PBST. After washing in PBST, sections were incubated for 4 h at 20 °C with Alexa-488 labeled goat anti-mouse IgG (1:1000, Molecular Probes, OR, USA) in BSA-PBST. After washing in PBS, the sections were mounted on glass slides with Fluoromount (Diagnostic BioSystems, CA, USA). GCaMP6s fluorescence images were acquired using a camera lucida attached to an epifluorescence microscope (BX51, Olympus, Tokyo, Japan) with 10 × , 20 × , and 40 × objective lenses.

### Statistics

No statistical method was used to predetermine sample sizes, but our sample sizes were similar to those of previous V1 imaging studies using macaque monkeys^7, 13^. Statistical analyses were conducted using MATLAB (2017b). The Tukey–Kramer test was performed on the responses to visual stimuli presented at 15 locations in the visual field contralateral to the recording hemisphere to locate the receptive fields. For signal time series before and after stimulus onset, two-tailed, two-sample *t*-tests were used to compare decoding performance from the original data with that from shuffled data. Correlation coefficients of stimulus responses between tracked ROI pairs and those between shuffled pairs were compared using two-tailed, two-sample *t*-tests. Correlations of OSI values between the tracked ROI pairs were calculated using the Pearson correlation. The two-sample Kolmogorov–Smirnov test was performed to verify whether the distributions of the differences in preferred orientations in the ROI pairs tracked across different recordings varied from the uniform and exponential distributions.

## Supplementary Information

Supplementary Information.

Supplementary Video 1.

Supplementary Video 2.

Supplementary Video 3.

## Data Availability

The data used in the analysis of this study are available from the corresponding author upon reasonable request. A reporting summary of this article is available in the Supplementary Information.

## Code availability

MATLAB codes used in the analysis of this study are available from the corresponding author upon reasonable request.
