# Supplementary material for: Microendoscopic calcium imaging of the primary visual cortex of behaving macaques
Source: Sci Rep. 2021 Aug 23;11:17021. doi: 10.1038/s41598-021-96532-z (PMC8382832; doi:10.1038/s41598-021-96532-z)
Supplement: Supplementary file 1 — Supplementary Information. [file 41598_2021_96532_MOESM1_ESM.docx]

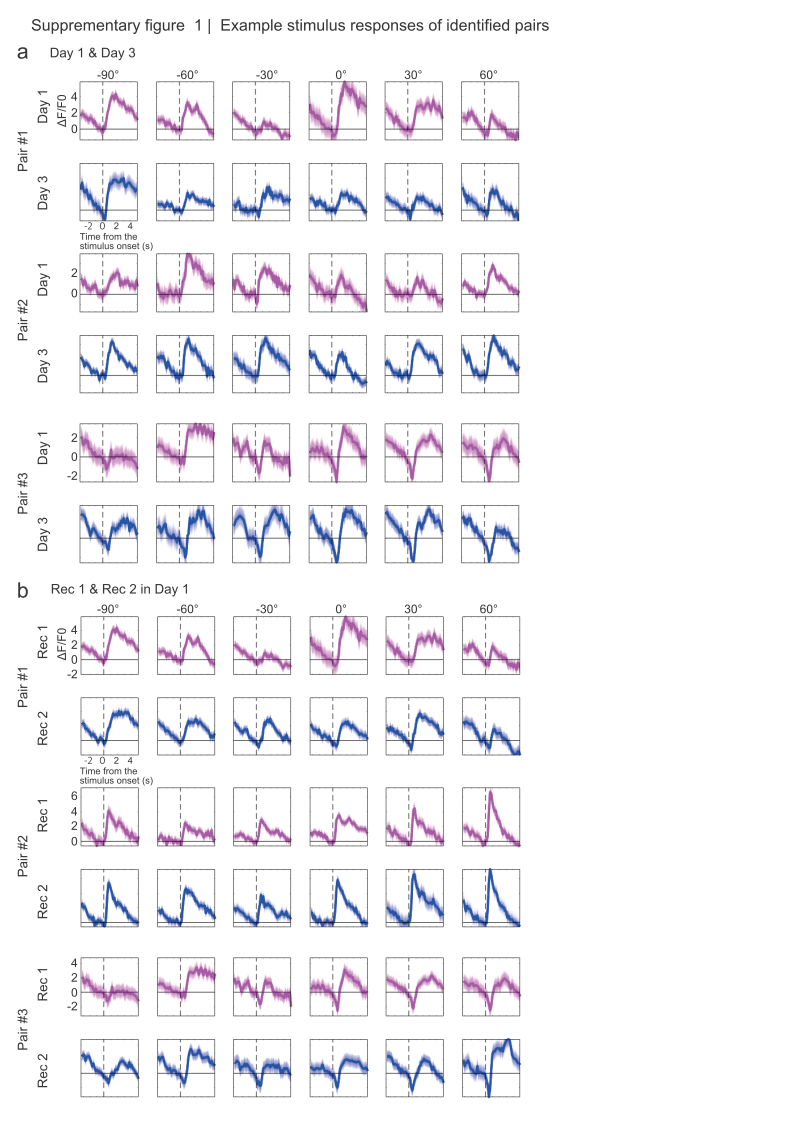


Supplementary figure 1 | Example stimulus responses of identified pairs

(a) The top three pairs with the highest correlation coefficients during stimulus responses between Day1 and Day3 (r = 0.893, 0.876, 0.874). These panels are arranged according to the orientation of the stimuli (columns) and pair and day (rows). Error areas: standard error of the mean. (b) The top three pairs with the highest correlation coefficients during stimulus responses between Rec1 and Rec3 on Day1 (r = 0.913, 0.913, 0.906). These panels are arranged according to orientation (columns) and pair and recording session (rows).


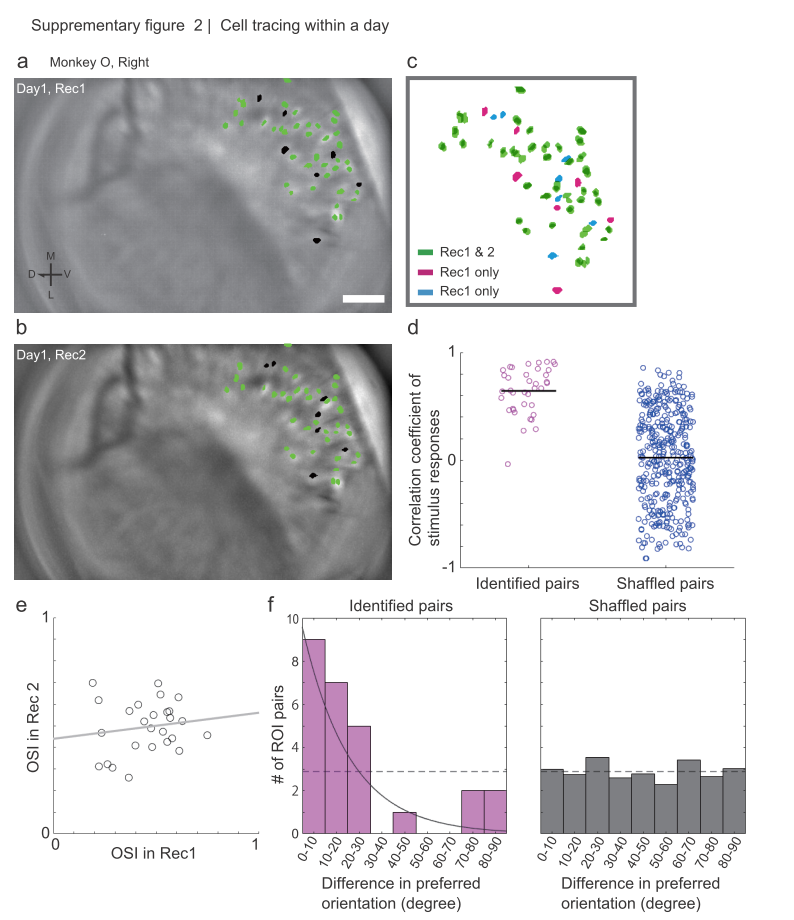


Supplementary figure 2 | Cell tracing within a day

Cell tracking was performed in two consecutive recordings of the same day (Rec1 and Rec2, both consisting of 120 trials). (a) ROI distribution map similar to that shown in Figure 5e. Green dots are identified as the same-colored dots in Rec2. The black dots represent ROIs that were not identified. (b) ROI distribution map of Rec2. Green dots are identified as the same-colored dots in Rec1. The black dots represent ROIs that were not identified. Scale bar: 100 µm. (c) ROIs detected in both Rec1 and Rec2 are shown in green, ROIs detected only on Rec1 are shown in magenta, and ROIs detected only on Rec2 are shown in cyan. (d) Correlation coefficients of fluorescence signals after stimulus presentation (0−2000 ms) between ROI pairs identified on Rec1 and Rec2 averaged over different orientations. The magenta dots represent identified pairs, and the blue dots represent shuffled pairs. (e) Scatter plot of OSI on Rec1 and Rec2 for identified pairs. The dots represent each identified ROI. (f) Histogram of the absolute values of the difference in preferred orientation between ROI pairs identified on Rec1 and Rec2. Zero indicates that the ROI showed the same value of preferred orientation on both recordings. The magenta bars in the left panel are the actual observed data, and the gray bars in the right panel are the shuffled data. The horizontal dashed line represents the chance level. The solid line represents an exponential curve fitted to the observed data. V, ventral; D, dorsal; M, medial; L, lateral.

Supplementary Movie 1. Video clip of a representative session recorded from the right hemisphere of Monkey O.

Supplementary Movie 2. Video clip of a representative session recorded from the left hemisphere of Monkey U.

Supplementary Movie 3. Video clip of a representative session recorded from the right hemisphere of Monkey U.
